# The Neuroprotective Effects of Spray-Dried Porcine Plasma Supplementation Involve the Microbiota−Gut−Brain Axis

**DOI:** 10.3390/nu14112211

**Published:** 2022-05-26

**Authors:** Cristina Rosell-Cardona, Concepció Amat, Christian Griñán-Ferré, Javier Polo, Mercè Pallàs, Anna Pérez-Bosque, Miquel Moretó, Lluïsa Miró

**Affiliations:** 1 Department of Biochemistry and Physiology, Faculty of Pharmacy and Food Sciences, Institute for Nutrition and Food Safety, Universitat de Barcelona (UB), 08028 Barcelona, Spain; cristina.rosell@ub.edu (C.R.-C.); camat@ub.edu (C.A.); anna.perez@ub.edu (A.P.-B.); mmoreto@ub.edu (M.M.); 2 Department of Pharmacology, Toxicology, and Medicinal Chemistry, Faculty of Pharmacy and Food Sciences, Institute of Neurosciences, Universitat de Barcelona (UB), 08028 Barcelona, Spain; christian.grinan@ub.edu (C.G.-F.); pallas@ub.edu (M.P.); 3 APC-Europe S.L.U., 08403 Granollers, Spain; javier.polo@apc-europe.com

**Keywords:** microbiota, Alzheimer’s disease, aging, dietary supplementation, spray-dried porcine plasma

## Abstract

Dietary supplementation with spray-dried porcine plasma (SDP) reduces the Alzheimer’s disease (AD) hallmarks in SAMP8 mice. Since gut microbiota can play a critical role in the AD progression, we have studied if the neuroprotective effects of SDP involve the microbiota−gut−brain axis. Experiments were performed on two-month-old SAMP8 mice fed a standard diet and on six-month-old SAMP8 mice fed a control diet or an 8% SDP supplemented diet for four months. Senescence impaired short- and long-term memory, reduced cortical brain-derived neurotrophic factor (BDNF) abundance, increased interleukin (*Il)-1β*, *Il-6*, and Toll-like receptor 2 *(Tlr2)* expression, and reduced transforming growth factor *β* (*Tgf-β)* expression and IL-10 concentration (all *p* < 0.05) and these effects were mitigated by SDP (all *p* < 0.05). Aging also increased pro-inflammatory cytokines in serum and colon (all *p* < 0.05). SDP attenuated both colonic and systemic inflammation in aged mice (all *p* < 0.05). SDP induced the proliferation of health-promoting bacteria, such as *Lactobacillus* and *Pediococcus*, while reducing the abundance of inflammation-associated bacteria, such as *Johnsonella* and *Erysipelothrix* (both *q* < 0.1). In conclusion, SDP has mucosal and systemic anti-inflammatory effects as well as neuroprotective properties in senescent mice; these effects are well correlated with SDP promotion of the abundance of probiotic species, which indicates that the gut–brain axis could be involved in the peripheral effects of SDP supplementation.

## 1. Introduction

Alzheimer’s disease (AD) is the most common type of dementia in elderly people. It is characterized by memory loss, the extracellular deposition of β-amyloid (Aβ) peptides and the aggregation of hyperphosphorylated tau (p-tau) protein, which is the main component of neurofibrillary tangles [1]. Neuroinflammation plays a critical role in AD because it can promote the accumulation of Aβ and p-tau [2]. There is currently no cure for this disease and the etiology underlying its progression remains unclear [3]. Although aging is the major risk factor for AD [4], many factors can influence its development, such as family history, susceptibility genes, and diet [5].

The gut microbiota, which comprises bacteria, archaea, fungi, and viruses that reside in the gastrointestinal tract, plays a key role in health and disease. In a healthy state, the microbiota is involved in the maintenance of the intestinal barrier, inhibition of pathogen adhesion to the epithelial surface, production of short-chain fatty acids (SCFAs), and regulation and maturation of the mucosal immune system [6]. During aging, the microbiota composition is altered, resulting in an increase in the abundance of pathogenic bacteria and a more pro-inflammatory microbiota profile. This type of microbial alteration, which is called dysbiosis, is associated with many intestinal diseases, such as inflammatory bowel disease and irritable bowel syndrome, but is also linked to extraintestinal inflammatory diseases such as asthma and obesity [7], as well as neurodegenerative diseases such as Parkinson’s disease and AD [8]. Furthermore, an altered microbiota composition and increase in pathogenic bacteria are associated with a disruption of the intestinal barrier [9]. Consequently, microbiota and their by-products can cross the intestinal barrier and activate toll-like receptors (TLR) and the immune system. This induces the release of pro-inflammatory cytokines and leads to an inflammatory state, which can promote neuroinflammatory responses and neurodegeneration in the central nervous system through the gut−brain axis [10].

The gut−brain axis is a network of connections involving neural, immune, endocrine, and metabolic pathways that enables communication between the gut and the brain. The concept of the microbiota−gut−brain axis has emerged because there is growing evidence of the role played by the microbiota in many neurological diseases [11]. Specifically, age-associated changes in the gut microbiota may play an important role in AD progression [12]. This interplay between the microbiota and the brain and their effects on AD are based on studies on germ-free animals or animals treated with antibiotics. Both models exhibit a reduction in working memory, cognitive function, synaptic plasticity, and an immaturity of microglia. Interestingly, behavioral and molecular changes in the brain and alterations in the gut microbiota have been reported in several AD mice models at different ages [13]. Notably, the use of probiotics, prebiotics, and fecal microbiota transplant alleviate neuroinflammation and improve memory [8]. Diet and dietary supplements are essential factors that can modulate the gut microbiota by increasing or decreasing the levels of some bacterial species and thereby modify the abundance of certain metabolites of microbial origin. In addition, they can play a vital role in protecting against AD via the gut−brain axis [14].

Spray-dried porcine plasma (SDP) is a functional protein source that contents many functional components such as albumin, immunoglobulins, transferrin, fibrinogen, growth factors and many other peptides that not only promote intestinal function but also have systemic effects beyond the intestine, such as on the respiratory and reproductive systems [15,16,17,18]. Using aged senescence-accelerated prone (SAMP8) mice, we have previously shown that dietary supplementation with SDP prevents cognitive decline and reduces neuroinflammation and oxidative stress in the central nervous system [19] and decreases the neuropathological hallmarks of AD [20]. Moreover, SDP has been widely studied for its anti-inflammatory properties in gut-associated lymphoid tissue and in several experimental models of chronic and acute inflammation [21,22]. SDP also exerts prebiotic effects by enhancing the levels of beneficial species that induce anti-inflammatory pathways in weaned mice [23]. Therefore, the present study aimed to elucidate if the microbiota−gut−brain axis is involved in the neuroprotective effects of SDP in aged SAMP8 mice, which is a well-established model of AD [24].

## 2. Materials and Methods

### 2.1. Animals, Experimental Design, and Diets

Male SAMP8 mice were obtained from Envigo (Bresso, Italy) and were kept at the animal facility of the Faculty of Pharmacy and Food Sciences of the Universitat de Barcelona under stable temperature and humidity conditions and a 12 h:12 h light/dark cycle. All animal experiments were performed following the Guide for the Care and Use of Laboratory Animals, and the protocols used in this study were approved by the Ethics Committee for Animal Experimentation of the Universitat de Barcelona and the Catalan government (ref. 337/19 and 11,128, respectively). Animals were housed individually and fed experimental diets (control (CTL) or SDP) for 4 months. SDP is a functional protein source obtained from the blood of healthy pigs intended for human consumption [25]. The diets were balanced for energy, lysine, methionine, and total nitrogen and were formulated to meet National Research Council requirements [26] for laboratory animals. The composition of the experimental diets is detailed in Rosell-Cardona et al. [20]. Mice were divided into three groups: 2M, 2-month-old mice fed standard commercial feed; 6M-CTL, 6-month-old mice fed Control diet for 4 months; and 6M-SDP, 6-month-old mice fed with SDP diet for 4 months.

### 2.2. Sample Collection

The day before animals were euthanized, their feces were collected under clean conditions and immediately frozen in liquid nitrogen. At the end of the experimental period, the mice were anesthetized by an intraperitoneal injection of ketamine:xylazine (100:10 mg/kg). Blood was obtained directly from cardiac puncture and tissue samples were taken from the brain cortex and colonic mucosa. All samples were quickly frozen at −80 °C until their use.

### 2.3. Open Field Test

The open field test (OFT) evaluates the locomotor activity and anxiety-like behavior of mice, based on their aversion to luminous and open spaces. It was performed as described by Puigoriol-Illamola et al. [27]. Briefly, mice were placed in the center of a white wooden box (50 × 50 × 25 cm) divided into central and peripheral zones (15 cm between the central zone and the wall). Mice explored the box for 5 min and each trial was recorded using a camera placed on the top of the box. The locomotor activity of the mice calculated as the sum of total distance travelled, the number of rearings, and the border stay duration were analyzed with SMART® v3.0 software (Harvard Bioscience, Holliston, MA, USA). Between each trial, the box was carefully cleaned with 70% ethanol.

### 2.4. Novel Object Recognition Test

The novel object recognition test (NORT) allows the evaluation of short- and long-term memory. It was carried out as described by Garcia-Just et al. [19]. Briefly, mice were placed in a 90-degree two-arm (25-cm long, 20-cm high, 5-cm wide) black maze. Mice were habituated to the maze for 10 min on three consecutive days. On the fourth day, the animals underwent a 10 min acquisition trial, in which they were placed in the maze in the presence of two identical objects situated at the end of each arm. During this trial, side preference and explorative index were measured. The first retention trial was performed two hours later to evaluate short-term memory. During this trial, one of the objects was replaced by a new one and the behavior of the mice was recorded for 10 min. The second retention trial, which measures long-term memory, was conducted 24 h after the acquisition trial. Another new object replaced the other new object in the previous trial. The mice were recorded for 10 min. The time that mice spent exploring the new object (NO) and the time that mice spent exploring the old object (OO) were measured from video recordings of each trial. To evaluate the cognitive performance, the discrimination index (DI) was calculated, which is defined as (NO − OO)/(NO + OO). A lower DI indicates less memory capacity.

### 2.5. Western Blot

Western blot was performed as described by Miró et al. [21]. Briefly, brain cortex samples were homogenized using a Polytron homogenizer (PRO Scientific Inc., Oxford, CT, USA) at 20,000 rpm in a lysis buffer containing 50 mM Tris-HCl, 150 mM NaCl, 2 mM ethylene-diamine-tetra-acetic acid (EDTA), 1% Triton X-100, 1 mM phenylmethylsulfonyl fluoride (PMSF), 1 mM dithiothreitol (DTT) and 2% (*v*/*v*) protease inhibitor cocktail (all from Sigma-Aldrich, St. Louis, MI, USA). Equal amounts of protein (50 μg) were separated on 10%–15% SDS-PAGE and transferred to polyvinylidene difluoride membranes (Bio-Rad, Hercules, CA, USA). The membranes were then blocked and incubated overnight at 4 °C with specific primary antibodies against nuclear factor kappa-light-chain-enhancer of activated B cells (NF-κB) p65 (ser536), total NF-κB (both obtained from rabbit and used at 1/1000, Cell Signaling Technology Inc., Danvers, MA, USA), brain-derived neurotrophic factor (BDNF, from rabbit and used at 1/500, Santa Cruz Biotechnology Inc., Dallas, TX, USA) and actin (from mouse and used at 1/10,000, Sigma-Aldrich). Next, the membranes were incubated with HRP-conjugated secondary antibodies (Sigma-Aldrich). Protein bands were visualized using the Clarity chemiluminescence detection kit and ChemiDoc XRS+ instrument (both from Bio-Rad) and quantified using the gel analyzer plug-in Gel Lanes Fit from ImageJ software [28].

### 2.6. Real-Time PCR

RNA was extracted from the brain cortex and colonic mucosa as described previously [20]. Total RNA was reverse-transcribed using the High-capacity cDNA Reverse Transcription Kit (Thermo Fisher Scientific, Waltham, MA, USA). Real-time PCR was performed on a MiniOpticon Real-Time PCR System (Bio-Rad). The primers used are shown in Supplementary Table S1. TaqMan gene expression assays (Applied Biosystems, Foster City, CA, USA) were used for *Occludin* (Mm0128968_m1) according to the manufacturer’s instructions. Product fidelity was confirmed by melting-curve analysis. Each PCR run included duplicates of reverse transcription for each sample and negative controls (reverse transcription-free samples, RNAfree sample). Target gene transcripts were quantified using *Hprt1* gene expression as reference and with the 2^−ΔΔCT^ method [29].

### 2.7. Quantification of Cytokines

Cytokine concentrations in serum and cortex samples were quantified using Bio-Plex Pro™ Cytokine, Chemokine and Growth Factor Assay kit (Bio-Rad), according to the manufacturer’s instructions.

### 2.8. Lipopolysaccharide Determination

Serum and cortical lipopolysaccharide (LPS) levels were determined using the Competition ELISA kit for LPS (Antibodies-online Inc., Atlanta, GA, USA), according to the manufacturer’s instructions.

### 2.9. Extraction and Purification of Total Genomic DNA

DNA was extracted as described previously by Moretó et al. [23]. Briefly, fecal samples were suspended in 4 M guanidine thiocyanate, 10% N-lauroyl sarcosine and 5% N-lauroyl sarcosine (all from Sigma-Aldrich). DNA was extracted by mechanical disruption of the microbial cell wall using Zirconia/silica beads (BioSpec Products, Bartlesville, OK, USA). The disruption was performed by shaking the mixture using the FastPrep®−24 instrument (MP Biomedicals, Solon, OH, USA). Then, polyvinylpolypyrrolidone was added (Sigma-Aldrich) and tubes were vortexed and centrifuged for 3 min at 12,000× *g*. The pellet was washed with TENP (50 mM Tris (pH 8), 20 mM EDTA (pH 8), 1% polyvinylpolypyrrolidone, 100 mM NaCl). DNA was precipitated by isopropanol and incubated with RNase (Qiagen, Venlo, The Netherlands). To precipitate nucleic acids, 3 M sodium acetate (Sigma-Aldrich) and absolute ethanol (JT Baker, Deventer, The Netherlands) were added. DNA was quantified using a NanoDrop ND-100 Spectrophotometer (Thermo Fisher Scientific).

### 2.10. 16S rDNA Gene Analysis

Extracted genomic DNA was processed and the variable V3 and V4 regions of the 16S rRNA gene were amplified. The primers to detect 16S rRNA were: 5′-TCGTCGGCAGCGTCAGATGTGTATAAGAGACAGCCTACGGGNGGCWGCAG-3′ (forward) and reverse 5′-GTCTCGTGGGCTCGGAGATGTGTATAAGAGACAGGACTACHVGGGTATCTAATCC-3′ (reverse). High-throughput sequencing was performed using the Illumina MiSeq platform (Illumina, San Diego, CA, USA) at the Genomics and Bioinformatics Service, Universitat Autònoma de Barcelona (Bellaterra, Spain).

### 2.11. Statistical Analysis

Results are presented as means ± standard error of the mean (SEM). All results were analyzed using GraphPad Prism® software v8 (GraphPad Software Inc., La Jolla, CA, USA). First, Grubb’s test was performed to detect outliers, in addition to Levene’s test to check the homogeneity of variance and the Shapiro–Wilk test to verify data normality for all groups. To compare groups, one-way ANOVA followed by the Fisher posthoc test was used for normally distributed data. In contrast, the Kruskal–Wallis test was used for non-normally distributed data. To analyze the evolution of body weight and feed consumption of the aged animals, the area under the curve was calculated for each animal and these values were compared using the t-student test. Differences were considered statistically significant when *p* < 0.05. Microbial data were analyzed with ANOVA test, followed by the Benjamini and Hochberg method that corrected *p* values (or *q* values) for the false discovery rate (FDR) for normally distributed data. In contrast, the Kruskal–Wallis test was used for non-normally distributed data. Differences were considered statistically significant when *q* < 0.1 [30].

## 3. Results

### 3.1. Body Weight and Food Intake

**Figure 1:**
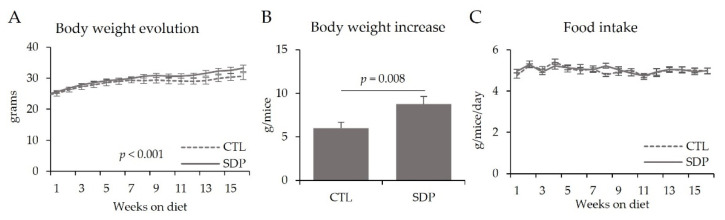
Body weight evolution (**A**), body weight increase (**B**) and food intake evolution (**C**) of SAMP8 mice during the experimental period. Results are expressed as mean ± SEM (*n* = 11–12 mice/group). Statistics: t-Student test.

Aged animals fed the SDP feed showed a higher rate of body weight gain than control (CTL) mice (*p* < 0.001; Figure 1A). At the end of the experimental period, body weight gain was greater in SDP mice than in CTL mice (*p* = 0.008; Figure 1B). Both groups of animals had similar food intake (Figure 1C).

### 3.2. Behavioral and Cognitive Tests

**Figure 2:**
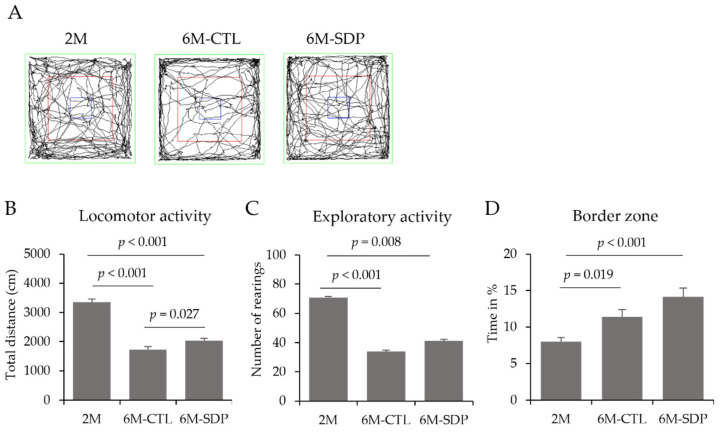
Open field test (OFT). Representative tracking maps of 2M, 6M-CTL, and 6M-SDP groups (**A**), locomotor activity (**B**), exploratory activity (**C**) and percentage of time in the border zone (**D**). Results are expressed as mean ± SEM (*n* = 11–12 mice/group). Statistics: ANOVA (Fisher multiple comparison test), and Kruskal–Wallis test.

AD is associated with behavioral and cognitive abnormalities, so we evaluated whether SDP could mitigate these alterations in aged SAMP8 mice. Representative tracking maps of the OFT of all groups are shown in Figure 1A. Aging reduced the locomotor activity of the mice (6M-CTL vs. 2M; *p* < 0.001; Figure 2B) and SDP attenuated this reduction (6M-SDP vs. 6M-CTL; *p* = 0.027). Furthermore, we showed reduced exploratory activity (6M-CTL and 6M-SDP, *p* < 0.001 and *p* = 0.008; Figure 2C), and increased anxiety-like behavior (6M-CTL and 6M-SDP, *p* = 0.019 and *p* < 0.001; Figure 2D) in both groups of aged mice, without differences due to SDP supplementation.

**Figure 3:**
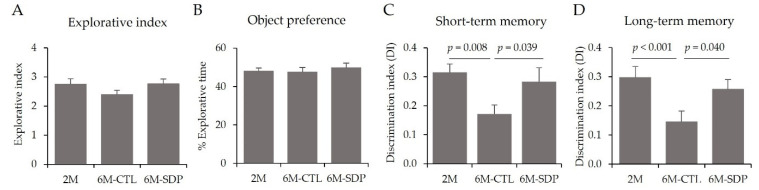
Novel object recognition test (NORT). Explorative index (**A**), object preference (**B**), short-term memory (**C**), long-term memory (**D**). Results are expressed as mean ± SEM (*n* = 11–12 mice/group). Statistics: ANOVA (Fisher multiple comparison test).

To assess the working memory of mice, they were evaluated using the NORT. During the familiarization phase, all groups had a similar explorative index and object preference (Figure 3A,B, respectively). Regarding short-term memory, aged mice had lower values of DI than younger mice (6M-CTL vs. 2M; *p* = 0.008; Figure 3C). SDP supplementation prevented this reduction (6M-SDP vs. 6M-CTL; *p* = 0.039), showing similar DI values to those of young mice. Regarding long-term memory, aged mice had a lower DI than the young group (6M-CTL vs. 2M; *p* < 0.001; Figure 3D). SDP supplementation prevented this reduction, as these mice showed higher DI values than aged mice (6M-SDP vs. 6M-CTL; *p* = 0.040).

### 3.3. Effects on the CNS

**Figure 4:**
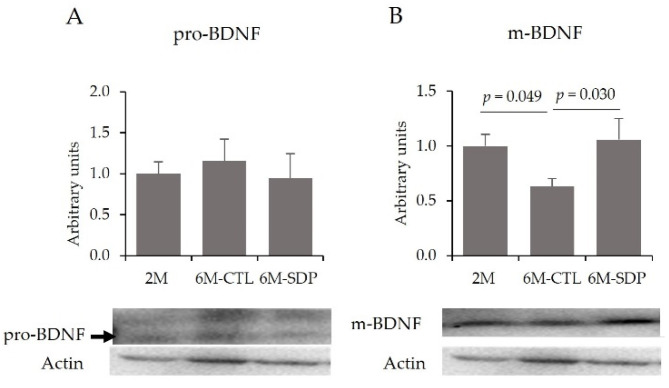
Abundance of BDNF precursor (pro-BDNF) (**A**) and mature BDNF (m-BDNF) (**B**). Results are expressed as mean ± SEM (*n* = 5−6 mice/group). Statistics: ANOVA (Fisher multiple comparison test), and chi-square test.

Molecularly, it has been well-established that SAMP8 presents changes in brain-derived neurotrophic factor (BDNF) pathway. Neither aging nor SDP supplementation changed the relative abundance of the precursor form of BDNF (pro-BDNF; Figure 4A) in the cortex. However, senescence reduced the abundance of mature form of BDNF (m-BDNF; 6M-CTL vs. 2M; *p* = 0.049; Figure 4B), which was prevented by SDP supplementation (6M-SDP vs. 6M-CTL; *p* = 0.030).

**Figure 5:**
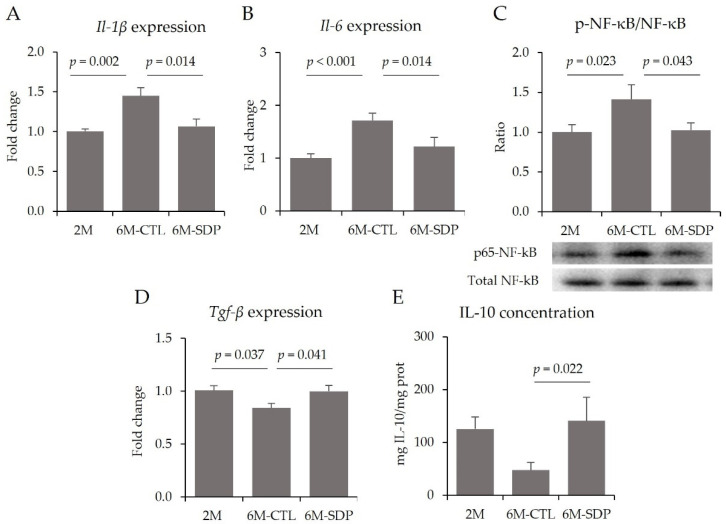
Neuroinflammation in cortical tissue. Gene expression of *Il-1β* (**A**) and *Il-6* (**B**), protein abundance of p65-NF-κB respect to total NF-κB (**C**), gene expression of *Tgf-β* (**D**), and concentration of IL-10 (**E**). Results are expressed as mean ± SEM (*n* = 8–9 mice/group). Statistics: ANOVA (Fisher multiple comparison test). Il, interleukin; NF-κB, nuclear factor kappa-light-chain-enhancer of activated B cells; Tgf-β, transforming growth factor-beta.

Gene expression of the pro-inflammatory cytokines Il-1β and Il-6 was increased in the cortex of aged mice (6M-CTL vs. 2M; *p* = 0.002 and *p* < 0.001, respectively; Figure 5A,B), as was the abundance of p65-NF-κB (6M-CTL vs. 2M; *p* = 0.023; Figure 5C). Supplementation with SDP reduced the gene expression of the pro-inflammatory cytokines (both *p* = 0.014) and attenuated the effect of aging on the abundance of p65-NF-κB (6M-SDP vs. 6M-CTL; *p* = 0.043). In addition, aged mice showed decreased gene expression of the anti-inflammatory cytokine Tgf-β compared with young mice (6M-SDP vs. 6M-CTL; *p* = 0.037; Figure 5D). SDP supplementation prevented this decrease (6M-SDP vs. 6M-CTL; *p* = 0.041) and also increased the concentration of IL-10 in the cortex of senescent mice (6M-SDP vs. 6M-CTL; *p* = 0.022; Figure 5E).

**Figure 6:**
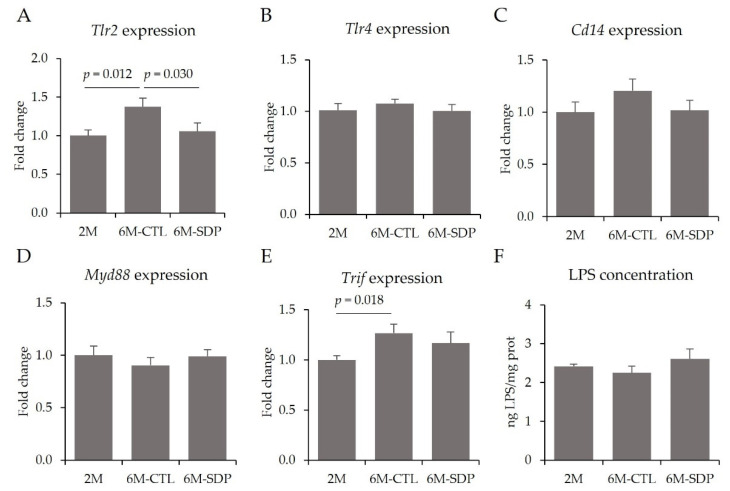
TLR signaling pathway in cortical tissue. Gene expression of *Tlr2* (**A**), *Tlr4* (**B**), *Cd14* (**C**), *Myd88* (**D**), *Trif* (**E**), and concentration of LPS (**F**). Results are expressed as mean ± SEM (*n* = 6–9 mice/group). Statistics: ANOVA (Fisher multiple comparison test). Cd14, cluster of differentiation 14; LPS, lipopolysaccharide; Myd88, myeloid differentiation factor 88; Tlr, toll-like receptor; Trif, toll-like receptor adaptor molecule 1.

Moreover, the gene expression of toll-like receptor 2 (*Tlr2*) was higher in senescent mice (6M-CTL vs. 2M; *p* = 0.012; Figure 6A), and SDP supplementation prevented this age-associated increase (6M-SDP vs. 6M-CTL; *p* = 0.030). On the other hand, no effects of aging or diet were observed for *Tlr4*, *Cd14*, and *Myd88* gene expression (Figure 6B–D, respectively). The gene expression of TIR-domain-containing adapter-inducing interferon-β (*Trif*) was increased in the cortex by senescence (6M-CTL vs. 2M; *p* = 0.018; Figure 6E). Conversely, the concentration of LPS in the cortex was not modified by aging or diet (Figure 6F).

### 3.4. Systemic Effects

**Figure 7:**
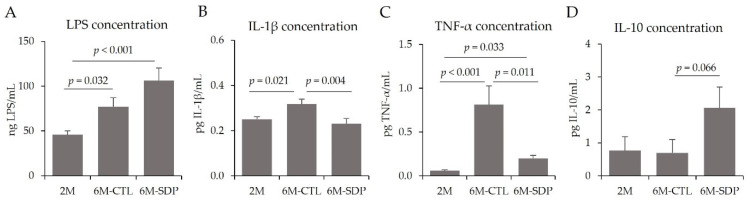
Systemic inflammation. Concentrations of LPS (**A**), IL-1β (**B**), TNF-α (**C**), and IL-10 (**D**). Results are expressed as mean ± SEM (*n* = 8–9 mice/group). Statistics: ANOVA (Fisher multiple comparison test). IL, interleukin; LPS, lipopolysaccharide; TNF-α, tumor necrosis factor-alpha.

Aging has been previously described to increase systemic inflammation in SAMP8 mice [29]. Of note, senescence increased systemic inflammation by augmenting the concentration of LPS (6M-CTL vs. 2M; *p* = 0.032; Figure 7A), and of the pro-inflammatory cytokines IL-1β and TNF-α in the serum of aged mice (6M-CTL vs. 2M; *p* = 0.021 and *p* < 0.001; Figure 7B,C, respectively), and SDP supplementation reduced the concentration of both cytokines in senescent mice (6M-SDP vs. 6M-CTL; *p* = 0.004 and *p* = 0.011, respectively). Furthermore, SDP supplementation tended to increase the serum concentration of the anti-inflammatory cytokine IL-10 (6M-SDP vs. 6M-CTL; *p* = 0.066; Figure 7D).

### 3.5. Gut Microbiota

**Figure 8:**
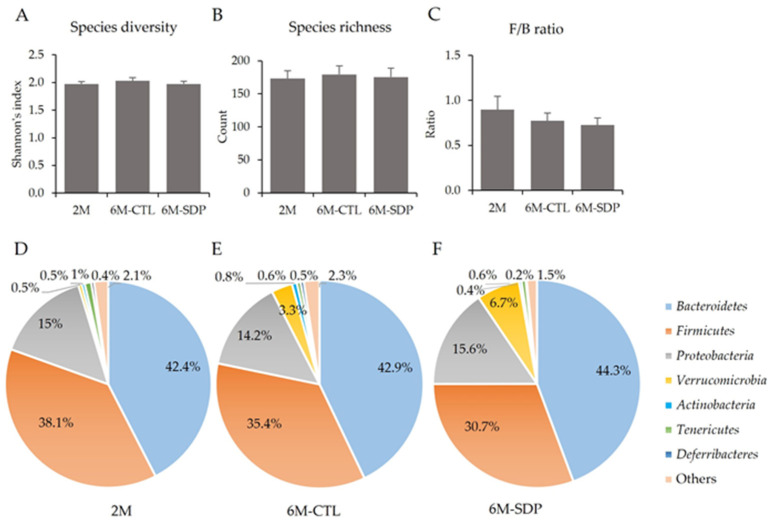
Microbial Shannon’s index (**A**), species richness (**B**), ratio between *Firmicutes*/*Bacteroidetes* (F/B) (**C**), and bacterial composition at the phylum level in the 2M group (**D**), 6M-CTL group (**E**), and 6M-SDP group (**F**). Results are expressed as mean ± SEM and as percentages (*n* = 10–11 mice/group). Statistics: ANOVA (Benjamini and Hochberg multiple comparison test).

The Shannon’s index, used as a reflection of the diversity of species in a community, was similar in the different groups (Figure 8A). The number of species in feces (species richness) was not modified by age or SDP supplementation (Figure 8B). The gut microbiota of these mice predominantly comprised *Bacteroidetes* (42.4%–44.3%; Figure 8D–F) and *Firmicutes* (30.7%–38.0%), which constituted the dominant phyla, and their ratio (F/B) was not modified by age or SDP supplementation (Figure 7C). They were followed by *Proteobacteria* (14.2%–15.5%), *Verrucomicrobia* (0.5%–6.7%), *Actinobacteria* (0.4%–0.8%), *Tenericutes* (0.6%–1.0%), *Deferribacteres* (0.2%–0.5%), and others. No significant differences could be attributed to age or SDP supplementation.

The effects of age and SDP supplementation at the family level are shown in Table 1. Senescence reduced the abundance of the families *Lactobacillaceae* and *Clostridiaceae*, from the Firmicutes phylum; *Sphingobacteriaceae*, *Flavobacteriaceae*, and *Prevotellaceae*, from Bacteroidetes phylum; and *Helicobacteraceae*, from the Proteobacteria phylum (6M-CTL vs. 2M; all *q* < 0.05); whereas increased *Erysipelotrichaceae*, from the Firmicutes phylum (6M-CTL vs. 2M; *q* < 0.05); and *Bacteroidaceae* and *Porphyromonadaceae*, from the Bacteroidetes phylum (6M-CTL vs. 2M; both, *q* < 0.1). On the other hand, SDP supplementation prevented the effect of aging on the *Lactobacillaceae* family and increased the relative abundance of the *Eubacteriaceae* family (6M-SDP vs. 6M-CTL; both *q* < 0.05).

**Figure 9:**
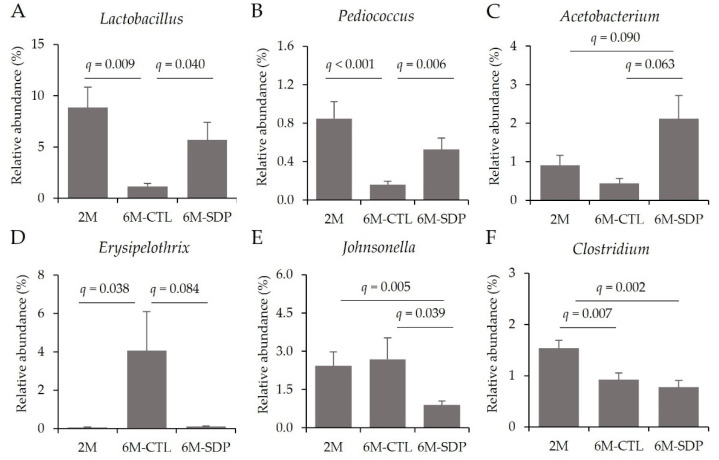
Fecal microbial composition at the genus level of the Firmicutes phylum. Relative abundance of *Lactobacillus* (**A**), *Pediococcus* (**B**), *Acetobacterium* (**C**), *Erysipelothrix* (**D**), *Johnsonella* (**E**), and *Clostridium* (**F**). Results are expressed as mean ± SEM (*n* = 10–11 mice/group). Statistics: ANOVA (False discovery rate, Benjamini and Hochberg multiple comparison test).

At the genus level and within the Firmicutes phylum, senescence reduced the relative abundance of the anti-inflammatory genera *Lactobacillus* and *Pediococcus* (6M-CTL vs. 2M; *q* = 0.009 and *q* < 0.001, respectively; Figure 9A,B) and SDP supplementation prevented these reductions (6M-SDP vs. 6M-CTL; *q* = 0.040 and *q* = 0.006, respectively). Furthermore, SDP supplementation increased the abundance of the acetyl-CoA producer *Acetobacterium* (6M-SDP vs. 6M-CTL; *q* = 0.063; Figure 9C). The abundance of the pathogenic bacteria *Erysipelothrix* was increased in aged mice compared with young mice (6M-CTL vs. 2M; *q* = 0.038; Figure 9D). SDP supplementation reduced it (6M-SDP vs. 6M-CTL; *q* = 0.084) and also reduced the relative abundance of the pathogenic bacteria *Johnsonella* (6M-SDP vs. 6M-CTL; *q* = 0.039, Figure 9E). Besides, aging reduced *Clostridium* abundance (6M-CTL vs. 2M; *q* = 0.007, Figure 9F), but SDP had no effects on its abundance.

**Figure 10:**
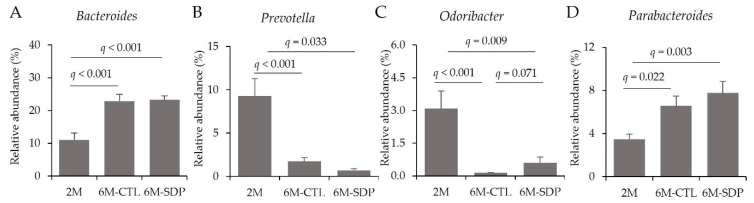
Fecal microbial composition at the genus level of the Bacteroidetes phylum. Relative abundance of *Bacteroides* (**A**), *Prevotella* (**B**) *Odoribacter* (**C**), and *Parabacteroides* (**D**). Results are expressed as mean ± SEM (*n* = 10–11 mice/group). Statistics: ANOVA (False discovery rate, Benjamini and Hochberg multiple comparison test).

At the genus level and considering the Bacteroidetes phylum, senescence increased *Bacteroides* abundance (6M-CTL vs. 2M; *q* < 0.001; Figure 10A), although no effect of SDP supplementation was observed. Aging reduced the relative abundance of *Prevotella* and SDP supplementation has no effect (6M-CTL vs. 2M; *q* = 0.013; Figure 10B). Senescence reduced levels of *Odoribacter* (6M-CTL vs. 2M; *q* < 0.001; Figure 10C) and SDP supplementation attenuated this effect (6M-SDP vs. 6M-CTL; *q* = 0.071). The *Parabacteroides* genus was increased in aged mice (6M-CTL vs. 2M; *q* = 0.022, Figure 10D) and there was no effect of SDP supplementation.

At the species level, aging reduced the abundance of several species of the Lactobacillus genus such as *L. taiwanensis*, *L. siliginis*, *L. antri* and *L. intermedius* as well as *Pediococcus argentinicus* (6M-CTL vs. 2M; all *q* < 0.05; Table 2), and SDP supplementation prevented this effect on *L. siliginis* levels (6M-SDP vs. 6M-CTL; *q* = 0.047). Aged mice showed an increase in some species of *Bacteroides spp.*, along with *Erysipelothrix muris* and *Parabacteroides gordonii* (6M-CTL vs. 2M; all *q* < 0.05), and a reduction in *Helicobacter mastomyrinus* and *Prevotella dentasini* (6M-CTL vs. 2M; both *q* < 0.05). SDP supplementation reduced the abundance of *Erysipelohtrix muris*, *Helicobacter mastomyrinus*, *Bacteroides denticanum*, and *Bifidobacterium choerinum* (6M-SDP vs. 6M-CTL; all *q* < 0.05) as well as *Parabacteroides gordonii* (6M-SDP vs. 6M-CTL; *q* < 0.1) and increased *Parabacteroides goldsteinii* (6M-SDP vs. 6M-CTL; *q* < 0.05).

### 3.6. Effects on Colon Tissue

**Figure 11:**
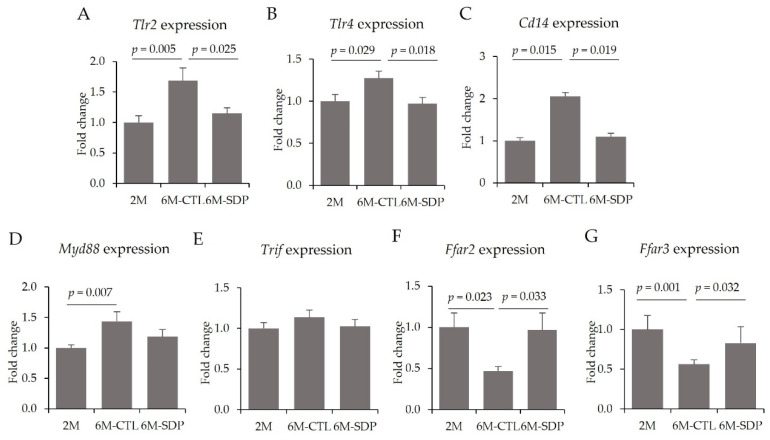
Gene expression of the TLR signaling pathway and SCFA receptors in colon mucosa. *Tlr2* (**A**), *Tlr4* (**B**), *Cd14* (**C**), *Myd88* (**D**), *Trif* (**E**), *Ffar2* (**F**), and *Ffar3* (**G**). Results are expressed as mean ± SEM (*n* = 9–11 mice/group). Statistics: ANOVA (Fisher multiple comparison test). Cd14, cluster of differentiation 14; Ffar2, free fatty acid receptor 2 (GPR43); Ffar3, free fatty acid receptor 3 (GPR41); Myd88, myeloid differentiation factor 88; Tlr, toll-like receptor; Trif, toll-like receptor adaptor molecule 1.

Gene expression analysis revealed that senescence increased the colonic expression of Tlr2 and Tlr4 (6M-CTL vs. 2M; *p* = 0.005 and *p* = 0.029; Figure 11A,B, respectively) and SDP supplementation prevented both increases (6M-SDP vs. 6M-CTL; *p* = 0.025, and *p* = 0.018, respectively). In addition, aging augmented the gene expression of *Cd14* in the colonic mucosa (6M-CTL vs. 2M; *p* = 0.015; Figure 11C), whereas SDP supplementation attenuated this increase (6M-SDP vs. 6M-CTL; *p* = 0.019). Likewise, *Myd88* gene expression was also increased in the colonic mucosa of aged mice compared with the young group (6M-CTL vs. 2M; *p* = 0.007; Figure 11D). No effect was observed of aging or SDP supplementation on *Trif* gene expression (Figure 11E).

Gene expression of *Ffar2* (which encodes GPR43) and *Ffar3* (which encodes GPR41) was reduced in the colon tissue of aged mice (6M-CTL vs. 2M; *p* = 0.023 and *p* = 0.001; Figure 11F,G, respectively), whereas SDP supplementation increased the expression of both genes (6M-SDP vs. 6M-CTL; *p* = 0.033 and *p* = 0.032, respectively).

**Figure 12:**
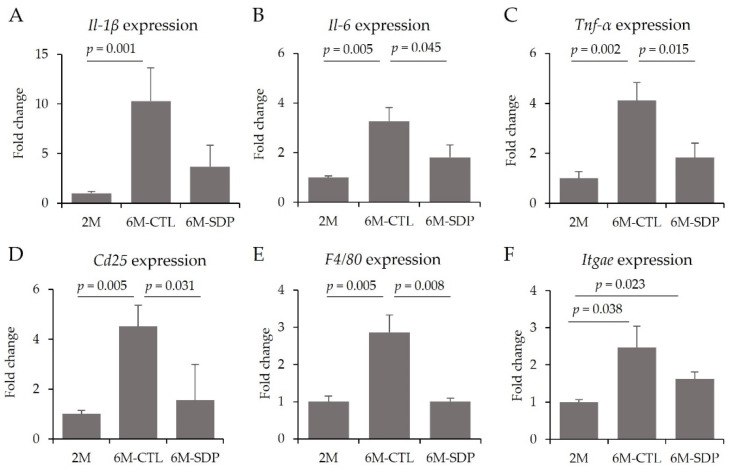
Inflammation in colon tissue. Gene expression of *Il-1β* (**A**), *Il-6* (**B**), *Tnf-*α (**C**), *Cd25* (**D**), *F4/80* (**E**), and *Itgae* (**F**). Results are expressed as mean ± SEM (*n* = 9−11 mice/group). Statistics: ANOVA (Fisher multiple comparison test) and Kruskal–Wallis test. Cd25, cluster of differentiation 25; Itgae, integrin alpha e; Il, interleukin; Tnf-α, tumor necrosis factor alpha.

In aged mice, the gene expression of pro-inflammatory cytokines (*Il-1β, Il-6, Tnf-α*) was augmented in the colonic mucosa (6M-CTL vs. 2M; *p* = 0.001, *p* = 0.005 and *p* = 0.002; Figure 12A–C, respectively), and SDP supplementation prevented the effects of aging on *Il-6*, and *Tnf-α* (6M-SDP vs. 6M-CTL; *p* = 0.045 and *p* = 0.015, respectively). Aging increased the gene expression of *Cd25* and *F4/80* (6M-CTL vs. 2M; both *p* = 0.005; Figure 12D,E, respectively), whereas SDP supplementation prevented these increases (6M-SDP vs. 6M-CTL; *p* = 0.031 and *p* = 0.008, respectively). *Itgae* was increased in control and SDP-treated senescent mice (*p* = 0.038 and *p* = 0.023, respectively; Figure 12F).

**Figure 13:**
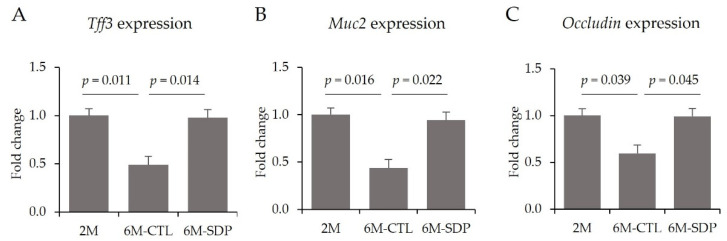
Intestinal barrier. Gene expression of *Tff3* (**A**), *Muc2* (**B**), and *Occludin* (**C**). Results are expressed as mean ± SEM (*n* = 9−11 mice/group). Statistics: ANOVA (Fisher multiple comparison test). Muc2, mucin 2; Tff3, trefoil factor 3.

Senescence also impaired the intestinal barrier by reducing the gene expression of *Tff3*, *Muc2*, and *Occludin* (6M-CTL vs. 2M; *p* = 0.011, *p* = 0.016, and *p* = 0.039; Figure 13A–C, respectively). SDP supplementation prevented all of these aging-associated changes (6M-SDP vs. 6M-CTL; *p* = 0.014, *p* = 0.022, and *p* = 0.045, respectively).

## 4. Discussion

There is consistent evidence supporting the ability of diet and dietary supplements to influence the progression of Alzheimer’s disease (AD) through the microbiota−gut−brain axis [14]. Mechanisms of communication between the gut microbiota and brain primarily involve the regulation of the immune system and the activation of neural and endocrine pathways by the gut microbiota or its by-products [31]. Dietary supplementation with SDP improves brain resilience against the neuropathological hallmarks of AD [15] and prevents the cognitive decline in SAMP8 mice [19]. Previous studies have shown that SDP supplementation has anti-inflammatory properties because it can modulate the mucosal immune response. Indeed, it can mitigate the harmful effects of *Staphylococcus aureus* enterotoxin B challenge in rodents [22] or *E. coli* infection in pigs [32]. Furthermore, SDP supplementation has prebiotic effects, raising the abundance of health-promoting bacteria in weaned mice [23], and weaned pigs [33]. Accordingly, in this study, we hypothesized that SDP promotes changes in the microbiota profile that could be involved in the neuroprotective effects of SDP.

As aforementioned, memory loss is one of the earliest reported symptoms in AD [1]. In our study, we observed age-associated short- and long-term memory deterioration, which is widely seen in SAMP8 mice [34]. We also found that SDP supplementation was protective against age-related cognitive decline [19]. The beneficial effect of SDP on cognition is related to a lower loss of synaptophysin in the brain [19], consistent with the fact that cognitive impairment can be due to synaptic dysfunction rather than neuronal loss [35]. Cognitive decline is also associated with reduced locomotor activity [36]. This age-related motor dysfunction is typically observed in SAMP8 mice [37], as we corroborated in aged SAMP8. For the first time, we have shown that SDP attenuated motor dysfunction in aged SAMP8 mice, similar to the increase in locomotor activity found in LPS-challenged C57BL/6 mice [38].

BDNF plays an essential role in synaptic plasticity, brain neuroprotection, and memory [39]. Of note, BDNF levels decreased in AD patients because Aβ peptides inhibit its maturation [40], leading to neurodegeneration and synaptic loss [41]. In our study, aged mice showed a lower abundance of mature form of BDNF, which correlates well with memory loss. SDP supplementation increased m-BDNF levels that could be responsible for the beneficial effect observed on memory retention. In addition, SDP supplementation reduced sAPPβ accumulation and Aβ_40_ concentration in the brain. In fact, SDP supplementation also reduced the expression of Bace-1, an essential enzyme in the amyloidogenic pathway [20], correlating the reduced activation of the amyloidogenic pathway with an increased abundance of the mature form of this neurotrophin. These results also agree with studies showing that dietary supplements and prebiotics can increase BDNF abundance in the brain and improve memory retention in adult rats [42].

Neuroinflammation and oxidative stress can worsen neurodegeneration and is considered a pathological hallmark of AD [2]. We have shown that SDP supplementation prevented neuroinflammation by diminishing the gene expression of the pro-inflammatory cytokines Tnf-α and Il-6 and increasing the levels of the anti-inflammatory cytokines Tgf-β and IL-10 in the cortex of aged mice. Moreover, in previous work, SDP supplementation also decreased the concentration of hydrogen peroxide in the cortex, reducing oxidative stress [19]. In addition, in this study, SDP supplementation also reduced gene expression of *Tlr2* as well as NF-κB activation. The expression of *Tlr2* is upregulated in brains of AD patients and in a mouse model of neurodegeneration [43]. Activation of TLR is not only essential for generating neuroinflammation, but this pathway also plays an essential role in promoting the amyloidogenic processing of Aβ [44]. Specifically, TLR2 and TLR4 are binding sites for Aβ, and the signaling of both receptors can activate microglia and induce the release of proinflammatory mediators [45]. The pathway activated through TLRs can be either Myd88-dependent or -independent pathways, both of which induce inflammatory cascades via activation of the transcription factor NF-κB [46]. Here, we observed that TLRs were activated in a Myd88-independent pathway because the *Trif* adaptor was increased in aged mice, while *Myd88* was not modified. Activation of NF-κB is associated with inflammatory responses and neurodegenerative diseases, such as in AD patients, where it induces the amyloidogenic pathway and tau phosphorylation [47]. The reduction in this signaling pathway observed in SDP-supplemented mice could be associated with the ability of SDP to decrease the amyloidogenic pathway and tau phosphorylation, as well as microglial activation [20].

Increasing evidence confirms the relationship between the microbiota and AD. The microbiota can modulate microglia maturation, regulate blood−brain barrier permeability and synaptic plasticity, or induce the formation of Aβ and neurofibrillary tangles [48]. Moreover, during aging, there is an alteration of the microbiota composition that contributes to the increase in the low-intensity inflammatory state present during senescence known as inflammaging. Dysbiosis also promotes LPS release and reduces the amount of beneficial bacterial metabolites such as SCFA [49]. In this study, the senescent mice exhibited a reduced abundance of beneficial species and an increase in pro-inflammatory bacteria. For instance, aging increased the abundance of the *Erysipelotrichaceae* family, and of the *Johnsonella* and *Erysipelothrix* genera, which are involved in inflammatory processes [50]. These pathogenic bacteria are more abundant in an AD mouse model [51] and may contribute to the cognitive decline in AD [52].

The SDP supplement mainly comprises peptides and proteins, which differentiates it from other conventional prebiotics [23]. Some of its compounds can resist digestion in the small intestine and reach the colonic lumen [53], where they can modify the colonic microbiota. SDP supplementation enhanced the abundance of probiotic genera with well-known anti-inflammatory properties at mucosal interfaces and reduced the abundance of pathogenic bacteria, such as the *Johnsonella* and *Erysipelothrix* genera. More specifically, at the species level, SDP prevented the effects of aging on the abundance of *Erysipelhotrix muris* and *Prevotella dentasini*. Furthermore, an SDP diet also enhanced the abundance of *Eubacteriaceae* family, which has health-promoting effects for the host, and is negatively correlated with dementia [54].

SDP supplementation promotes the abundance of the *Lactobacillaceae* family, as well as different health-promoting genera within it, such as *Lactobacillus* and *Pediococcus*. These effects of SDP on *Lactobacillus* have been also observed in other mice strains [23] and in pigs [33]. Furthermore, oral administration of *Lactobacillus* bacteria prevents cognitive decline in both humans and rats [55] and improves memory retention in aged SAMP8 mice [56], in addition to increasing BDNF levels in the rodent brain [57]. These observations are consistent with our findings regarding augmented BDNF abundance and prevention of cognitive decline. Furthermore, *Lactobacillus* exerts anti-inflammatory effects by increasing plasma IL-10 levels [57], which is also increased in SDP-supplemented senescent mice.

SDP supplementation also augmented the abundance of other bacterial genera of the Firmicutes phylum, such as *Acetobacterium*, which is an acetyl-CoA producer [58]. Increased acetyl-CoA levels improve cognitive function during aging [59] and acetyl-CoA is the main precursor of butyrate [60]. SCFAs, especially butyric acid, can inhibit the expression of the cytokines *Il-6*, *Il-1β*, and *Tnf-α*, thereby exerting anti-inflammatory effects [61]. SCFAs can mediate cellular functions by activating cell surface G protein-coupled receptors (GPCR), such as *Ffar2* and *Ffar3*, which are expressed on epithelial and immune cells [31]. Here, SDP supplementation increased the gene expression of *Ffar2* and *Ffar3*, which would suggest that some of the anti-inflammatory and neuroprotective effects of SDP may be mediated through SCFA. These byproducts produced by the microbiota may circulate to the brain, modulating the degree of immune system activation and regulating microglia maturation [31], and could therefore be a link between the gut microbiota and the reduction in neuroinflammation.

Aging-related dysbiosis promotes the production of LPS and the increase of the intestinal barrier permeability, which allows the translocation of bacteria and their by-products into the bloodstream [62]. Recent studies have revealed that increased intestinal permeability induces systemic inflammation, impairing the blood−brain barrier function, triggering neuroinflammation and cognitive dysfunction [8,63]. In the present study, it was observed that aged mice showed a lower expression of genes related to the production and maintenance of the mucus layer (*Tff3* and *Muc2*) and to the epithelial barrier tightness (*Occludin*) and, as consequence of the impaired intestinal barrier, the serum LPS concentration was elevated in aged mice. Indeed, AD patients show higher plasma LPS concentration, which may promote the development of the amyloid pathology [64]. In contrast, dietary supplementation with SDP prevented the age-associated changes in the gene expression of *Occludin*, *Muc2* and *Tff3*. These effects of SDP may be related to the effects observed on the microbiota, since *Lactobacillus* bacteria enhance the intestinal barrier, and reduce colon permeability [65], promoting mucus secretion, providing antimicrobial protection and preventing the translocation of pathogenic bacteria [66]. Indeed, this ability of SDP to improve intestinal barrier tightness has been observed in different experimental models of intestinal inflammation, both acute [67] and chronic [21] inflammation.

These effects of aging on intestinal barrier function are accompanied by colonic mucosal inflammation, and a recruitment of activated T cells (*Cd25*) and macrophages (*F4/80*). Bacteria can activate immune cells through TLR receptors. This is consistent with increased expression of *Tlr2* and Tlr4 in the colonic mucosa. Activation of the immune response results in systemic inflammation which is also common along aging [68]. In this work, the systemic concentration of pro-inflammatory cytokines (e.g., IL-1β and TNF-α), increased with aging. Patients with AD show elevated concentrations of circulating IL-1β and TNF-α [69], which are considered as peripheral biomarkers for AD [70]. Indeed, chronic systemic inflammation induces AD-like neuropathology in mice [63]. Various factors of the inflammatory cascade can circulate to the brain, cross the altered blood−brain barrier [10], and activate microglia, thereby altering cognitive function [48]. Dietary supplementation with SDP showed immunomodulatory effects by reducing the intensity of the immune response in the colon. The SDP diet decreased the gene expression of the innate receptors *Tlr2* and *Tlr4* and the recruitment of activated T cells and macrophages, as well as the expression of the pro-inflammatory cytokine *Tnf-α* and *Il-6.* This effect of SDP is relevant because immune activation and the release of pro-inflammatory cytokines can disassemble tight-junction proteins increasing epithelial permeability [71] and promoting and perpetuating intestinal inflammation [72]. SDP supplementation not only attenuated the immune response in the colon, but also systemically, by reducing the concentration of pro-inflammatory cytokines (TNF-α and IL-1β) in the serum as well as by increasing the concentration of the anti-inflammatory cytokine IL-10. The SDP induced reduction in systemic inflammation is consistent with previous results in aged mice [73].

## 5. Conclusions

**Figure 14:**
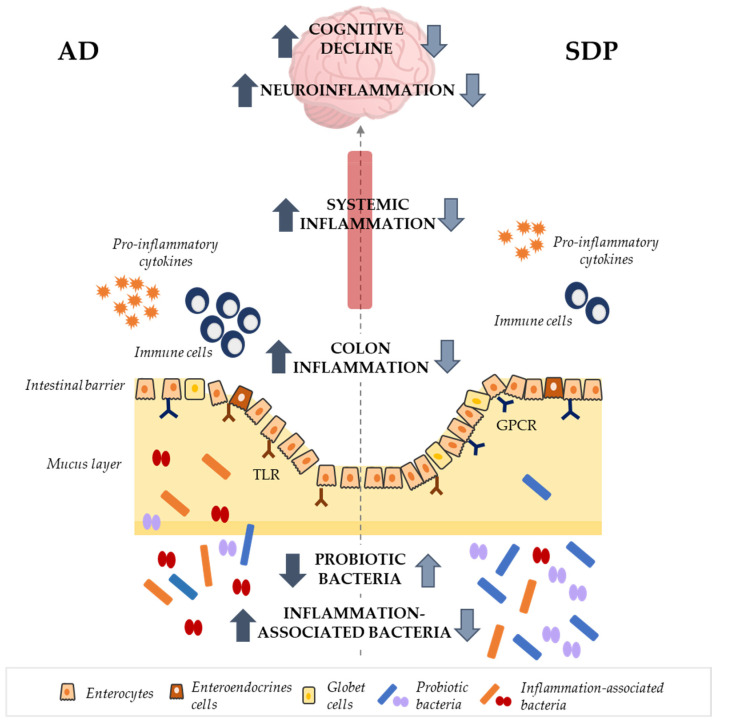
Schematic integrative representation of the effects of SDP supplementation on the gut−brain axis in an AD mouse model. Dietary supplementation with SDP promotes the growth of probiotic genera and reduces the levels of pathogenic genera. This is accompanied by a reduction in the intestinal permeability and local and systemic immune and inflammatory responses. These effects culminate in a reduction in neuroinflammation and an amelioration of the cognitive decline. AD, Alzheimer’s disease; GPCR, G protein-coupled receptors; SDP, spray-dried porcine plasma; TLR, toll-like receptor.

In conclusion, dietary supplementation with SDP increases the abundance of probiotic species in the gut and reduces the local and systemic inflammatory responses. This attenuation of the immune response results in reduced neuroinflammation, improving cognitive performance in senescent animals (Figure 14). Taken together, this study shows that SDP supplementation has prebiotic effects and suggests that the microbiota−gut−brain axis could play a role in the neuroprotective effects of SDP.

## Figures and Tables

**Table 1:** Bacterial composition at family level.

					*q*		
Phylum	Family	2M	6M-CTL	6M-SDP	2M 6M-CTL	2M 6M-SDP	6M-CTL 6M-SDP
*Firmicutes*	*Lactobacillaceae*	10.1 ± 2.2	1.3 ± 0.3	6.6 ± 2.0	0.003	NS	0.019
	*Eubacteriaceae*	0.8 ± 0.2	0.4 ± 0.1	2.0 ± 0.6	NS	NS	0.033
	*Erysipelotrichaceae*	0.1 ± 0.01	3.9 ± 1.9	0.5 ± 0.2	0.029	NS	NS
	*Lachnospiraceae*	20.1 ± 2.2	23.2 ± 2.2	17.6 ± 1.7	NS	NS	NS
	*Clostridiaceae*	6.7 ± 0.6	4.0 ± 0.4	4.7 ± 0.7	0.008	0.030	NS
	*Ruminococcaceae*	4.6 ± 0.5	3.6 ± 0.5	3.8 ± 0.6	NS	NS	NS
*Bacteroidetes*	*Bacteroidaceae*	10.2 ± 1.9	21.2 ± 1.9	21.8 ± 2.0	<0.001	<0.001	NS
	*Porphyromonadaceae*	8.0 ± 1.3	11.3 ± 1.0	12.1 ± 1.2	0.082	0.060	NS
	*Sphingobacteriaceae*	5.5 ± 0.6	3.8 ± 0.3	4.3 ± 0.3	0.036	NS	NS
	*Flexibacteraceae*	0.3 ± 0.1	0.5 ± 0.1	0.4 ± 0.1	NS	NS	NS
	*Flavobacteriaceae*	4.2 ± 0.6	2.2 ± 0.3	2.1 ± 0.4	0.005	0.005	NS
	*Odoribacteriaceae*	3.2 ± 0.7	1.9 ± 0.3	2.4 ± 0.5	NS	NS	NS
	*Prevotellaceae*	9.2 ± 2.2	1.6 ± 0.4	0.6 ± 0.2	0.018	0.017	NS
*Proteobacteria*	*Desulfovibrionaceae*	1.9 ± 0.4	2.6 ± 0.4	3.1 ± 0.4	NS	NS	NS
	*Enterobacteriaceae*	0.6 ± 0.1	0.5 ± 0.1	0.6 ± 0.1	NS	NS	NS
	*Helicobacteriaceae*	3.6 ± 0.7	1.4 ± 0.3	0.8 ± 0.3	0.004	<0.001	NS
*Verrucomicrobia*	*Verrucomicrobiaceae*	0.03 ± 0.01	3.2 ± 1.6	8.1 ± 1.9	NS	0.016	NS
*Actinobacteria*	*Bifidobacteriaceae*	0.05 ± 0.01	0.5 ± 0.3	0.05 ± 0.01	NS	NS	0.069

Results are expressed as percent of the total population at family level (mean ± SEM; *n* = 10–11 mice). Statistics: ANOVA (False discovery rate, Benjamini and Hochberg multiple comparison test). NS: non-significant.

**Table 2:** Effects of age and SDP on bacterial composition at species level.

					*q*		
Family	Species	2M	6M-CTL	6M-SDP	2M	2M	6M-CTL
					6M-CTL	6M-SDP	6M-SDP
*Lactobacillaceae*	*Lactobacillus hayakitensis*	5.1 ± 1.7	0.9 ± 0.2	2.0 ± 0.6	NS	NS	NS
	*Lactobacillus taiwanensis*	1.9 ± 0.7	0.2 ± 0.1	0.5 ± 0.3	0.003	0.070	NS
	*Lactobacillus siliginis*	1.8 ± 0.5	0.3 ± 0.06	0.8 ± 0.2	0.035	0.075	0.047
	*Lactobacillus antri*	1.1 ± 0.3	0.1 ± 0.02	0.3 ± 0.2	0.018	0.016	NS
	*Lactobacillus intermedius*	0.5 ± 0.2	0.1 ± 0.03	0.3 ± 0.1	NS	NS	NS
	*Pediococcus argentinicus*	0.5 ± 0.1	0.08 ± 0.04	0.2 ± 0.05	0.009	NS	NS
*Erysipelotrichaceae*	*Erysipelhotrix muris*	0.1 ± 0.02	8.9 ± 3.8	0.2 ± 0.1	0.020	NS	0.046
*Prevotellaceae*	*Prevotella dentasini*	16.0 ± 3.7	1.6 ± 0.4	0.7 ± 0.2	0.005	0.005	0.044
*Bacteroidaceae*	*Bacteroides xylanisolvens*	2.9 ± 0.4	6.6 ± 1.2	8.7 ± 1.3	0.031	0.002	NS
	*Bacteroides rodentium*	2.7 ± 0.6	3.2 ± 0.9	5.6 ± 1.3	NS	NS	NS
	*Bacteroides acidifaciens*	0.5 ± 0.1	4.1 ± 0.7	4.6 ± 0.9	0.001	0.001	NS
	*Bacteroides sartorii*	0.3 ± 0.1	1.1 ± 0.3	1.3 ± 0.1	0.011	0.002	NS
	*Bacteroides denticanum*	0.9 ± 0.1	6.5 ± 1.1	3.1 ± 0.6	0.001	0.009	0.015
*Porphyromonadaceae*	*Parabacteroides goldsteinii*	4.9 ± 0.6	6.0 ± 0.7	10.6 ± 1.3	NS	0.005	0.013
	*Parabacteroides gordonii*	0.0 ± 0.0	3.6 ± 0.8	1.7 ± 0.7	<0.001	0.014	0.070

Results are expressed as percent of the total population at species level (mean ± SEM; *n* = 10–11 mice). Statistics: ANOVA (False discovery rate, Benjamini and Hochberg multiple comparison test). NS: non-significant.

## Data Availability

The datasets generated during and/or analyzed during the current study are available from the corresponding author on reasonable request.

## Supplementary Materials

The following supporting information can be downloaded at: https://www.mdpi.com/article/10.3390/nu14112211/s1, Table S1: Primers used for Real-Time PCR.
