# Supplementary material for: The Neuroprotective Effects of Spray-Dried Porcine Plasma Supplementation Involve the Microbiota−Gut−Brain Axis
Source: Nutrients. 2022 May 26;14(11):2211. doi: 10.3390/nu14112211 (PMC9183112; doi:10.3390/nu14112211)
Supplement: Supplementary file 1 [file nutrients-14-02211-s001.zip › nutrients-1648814-supplementary.pdf]

Supplementary Table S1. Primers used for Real-Time PCR.

Primer	Forward (5′-3′)	Reverse (5′-3′)	Size (bp)
Cd14	TCGCTCAACTTTTCCTGCGA	ATCAGTCCTCTCTCGCCCAA	95
Cd25	TGGCAACACAGATGGAGGAA	CGTTAGGTGAATGCTTGGCG	124
F4/80	AACATGCAACCTGCCACAAC	TTCACAGGATTCGTCCAGGC	110
Ffar2	TAGCTCCCCGGTTTTGGTAG	AGTCTGGGGTCATTCTCCTTG	96
Ffar3	CCAGTTGTCCAATACTCTGCATC	CACGAGGAACACCAACAGGTA	99
Hprt1	TGGATACAGGCCAGACTTTGTT	TGGATACAGGCCAGACTTTGTT	163
Il-1β	GGTCAAAGGTTTGGAAGCAG	TGTGAAATGCCACCTTTTGA	94
Il-10	GGCGCTGTCATCGATTTCTCCCC	TGGCCTTGTAGACACCTTGGTCTT	102
Il-6	TGTGAAATGCCACCTTTTGA	GGTCAAAGGTTTGGAAGCAG	109
Itgae	TGACAAAGACTCAGGACCACAC	ATAGCACAGACCACTGAATGCC	106
Muc2	TGCTGCTGACGAGTGGTTGGTG	CGGACGCTTGGTGGTGAGGC	76
Myd88	TGCCAGCGAGCTAATTGAGAA	TGCCAGCGAGCTAATTGAGAA	130
Tff3	GCCCTCTGGCTAATGCTGTT	CTTGGAGACAGGACGCCAACGTA	80
Tgf-β	CAGGGTGAAGGGGAAAACTC	AGTTCGGTCATTAGTCTCGC	204
Tlr2	GTTTCTGAGTGTAGGGGCTTCA	CATCCTCTGAGATTTGACGCTTTG	122
Tlr4	GCTTGAATCCCTGCATAGAGGTAG	GAAGAAGGAATGTCATCAGGGACT	91
Tnf-α	CCACCACGCTCTTCTGTCTAC	AGGGTCTGGGCCATAGAACT	103
Trif	TAACACACCGCTGGACACTC	ACTGATGGAGGCCAGCTTA	85

Cd, cluster of differentiation; Ffar2, free fatty acid receptor 2 (GPR43); Ffar3, free fatty acid receptor 3 (GPR41); Hprt1, hypoxanthine phosphoribosyl transferase 1; Itgae, integrin alpha e; Il, interleukin; Muc2, mucin 2; Myd88, myeloid differentiation factor 88; Tff3, trefoil factor 3; Tgf-β, transforming growth factor-beta; Tlr, toll-like receptor; Tnf-α, tumor necrosis factor alpha; Trif, toll-like receptor adaptor molecule 1.
